# The Havoc of Two Storms

**Published:** 1913-05

**Authors:** G. Henri Bogart

**Affiliations:** Paris, Ill.


### The Havoc of Two Storms.

BY G. HENRI BOGART, M. D., PARIS, ILL.

Over two States swept a cyclonic twisted tornado, followed by raging, turbid torrents, storm and flood driving a tandem team of death and devastation, in whose wake raced the fire fiend and foul famine, hand in hand, the while pale Pestilence shouted her vile halloo behind them. The pall of horror hung over all the nation because of the cataclysm, the press gave aggregated acres of space to the details of the disaster, while the people lined up in front of the news stands, clamoring for the latest information. And this, too, when there was no lesson to be learned from the recital; when no foresight nor bravery could have averted one iota of the horror, and the only purpose of the readers was to minister to the natural sympathies of the human heart for fellows in misery.

At the same time, there was, and is, food for deep thought in this period when a national catastrophe has opened the portals of the soul. Comparisons that tell us of our own shortcomings may not be pleasant, but it is a certain fact that the toll of death and sorrow, and suffering is not a tithe of that which comes from the daily demands that are made on the lives and happiness of humanity by wholly preventable menaces, which we—the most of us—accept as a mere matter of course, if, indeed, we bestow a passing thought upon it.

In the storm-swept area there annually die more babies than the total toll of the disaster—babies who die because of the ignorance and carelessness of loving, doting mothers who would gladly have laid down their own lives that their darling pledges of love might have been saved, and who wonder in their empty desolation of heart bitterness that Providence could be so cruel; for those mothers enter upon the holy function of motherhood with no preparation and no knowledge; they entered the married relation with absolute lack of information—only the foul misrepresentations that came to the curious girl in an indirect manner—because it is not nice to think, much less to talk, about such things.

She had been taught, oh, so carefully, to dress, to sing and to play, to cook or at least to direct her household, to know literature and science—everything, except the highest, best, holiest element of her being, that of maternity.

It had been held in her education that "Ignorance is innocence," and any attempt to teach her of herself and her sacred purpose in living had been frowned upon, so much so that the majesty of the law would have been invoked to punish him who would have attempted to give to her the information of most worth.

And so the slaughter of the innocents goes merrily along—babes offered to the Moloch of prudery.

Annually, in the same territory as that which the elements devastated, there die from preventable diseases far greater numbers than were offered to the storm's fury.

Their deaths are more tragic and carry a greater aggregate of suffering for loved ones, and for the victims as well, for the death in the tornado or in the whirling, swirling flood was comparatively quick and merciful; and when the men who know and who seek to stay the awful waste would act, they are made the objects of distrust and abuse.

Annually, the twin blots on civilization, the alcohol and the social evils, cost more in mere dollars and cents than is the total of the property destroyed by the great calamity.

"It is not nice, Doctor, for you to speak of the social evil; you should be ashamed of yourself for even suggesting such a thing," says the prude; but when we investigate this damnable thing that is poisoning our nation and in addition to its financial cost, is piling an incalculable burden of woe and misery upon the innocent, we are appalled.

Lyman Beecher said: "We must educate, we must educate, or we must perish."

It is well that the women of the nation are awakening to the gravity of the conditions that hang like a huge cobra over them, over their homes, their happiness, and over their unborn children.

Then, while the mind is open and receptive to the horror of the great storm of 1913, let us pause and think.

In God's name, the peril is present and active enough that its ravages should not be concealed; it is growing, for the total consumption of alcohol grows greater each year, and as for its cognate and greater evil, the supply of victims of lust is not adequate to fill the demand, and the trail of the white slave demon was found in the wake of the storm, seeking to entrap those girls whom sorrow and want had rendered frantic by lying offers of work in some distant city, where they were to be driven into the cesspool. Women of America! Prove your womanhood by helping to drive this hell that menaces the best of your sex into the oblivion that will protect the coming generation, for you know not who of your own dear ones may be victims.

